# Supplementary material for: Communicating astrobiology and the search for life elsewhere: Speculations and promises of a developing scientific field in newspapers, press releases and papers
Source: PLoS One. 2025 Jul 29;20(7):e0328766. doi: 10.1371/journal.pone.0328766 (PMC12306777; doi:10.1371/journal.pone.0328766)
Supplement: S2 Appendix — (DOCX) [file pone.0328766.s002.docx]

**Appendix II**

**Codebook – Comparative framework (paper, press release, news article)**

**The portrayal of the search for life elsewhere in the media: speculations and expectations**

This codebook is designed to yield a clearer picture of the communication of research results related to the search for evidence of life beyond Earth by comparing three types of sources of scientific information: the original paper, the press release and the newspaper article. We will pay close attention to the similarities and differences among these sources in the portrayals of research results related to the search for life elsewhere (SLE).

Our goals are: 1. to get data to help us understand what are, in the science communication ecosystem, the main sources of speculations about the existence of life beyond Earth and of promises about the potential discovery of life elsewhere; 2. to understand how common the amplifications of these speculations and promises are in the public discourse; and 3. to understand how frequent misrepresentations of research results related to the SLE are in the public discourse.

We hope to provide the means to distinguish when the science communication ecosystem is providing the public with reasonable and necessary context to scientific research related to the SLE and when it’s amplifying the significance or the impact of a research result for the SLE by trying to anticipate its outcomes (e.g. portraying a result as just another step in an inevitable buildup to the discovery of life beyond Earth).

Regardless of what the current state of scientific knowledge suggests about the prevalence of life in the universe – the sheer number of potential planetary systems and the fact that complex organic molecules are being found in molecular clouds point to a possible positive outcome of the SLE – the coder must assume a position of agnosticism towards life beyond Earth and the search for it. We don’t know if life exists beyond our planet and we don’t know if we are ever going to reach a definite answer to the question about the plurality of inhabited worlds.

This instrument is also not designed to yield any data about the scientific results themselves – we’re not asking questions about the validity and the significance of the research results. We are strictly looking for the representations of these particular research results and the portrayals of the SLE as a scientific enterprise.

**Key concepts from astrobiology appear in the examples and instructions for coding.**

**Definitions from the *Encyclopedia of Astrobiology* (Gargaud, 2020), except for *Technosignatures* (Kaufman, 2018) and the section about conditions and ingredients for life (Domagal-Goldman & Wright et al, 2016).**

Abiogenesis: “Since the end of the nineteenth century, evolutive abiogenesis means increasing complexity and evolution of matter from inert to living state in the abiotic context of evolution of primitive Earth.” (Tirard, 2015).

Biogenicity: “Biogenicity refers to any chemical and/or morphological signature preserved over a range of spatial scales in rocks, minerals, ice, or dust particles that are uniquely produced by past or present organisms. This includes elemental and isotopic signatures diagnostic of life, which cannot be formed by purely abiotic processes.” (McLoughlin, 2015).

Biosignature, biomarker: “Biomarkers or “markers of life” or “traces of life” include chemical, morphological, sedimentary, or isotopic processes or structures that are biogenic and could be detected to infer the past or present presence of life.” (Javaux, 2015).

Exoplanet: “Exoplanets, also known as extrasolar planets, are planets beyond the solar system, orbiting around stars other than the Sun.” (Latham & Haghighipour, 2015).

Exomoon: “An exomoon is a natural satellite (as opposed to an artificial satellite) of an extrasolar planet.” (Haghighipour, 2015).

Extremophile: “Life is influenced by physical parameters such as temperature, pH, salinity, pressure, etc. Extremophiles are organisms that thrive in ecosystems where at least one physical parameter is close to the known limits of life with respect to this parameter. While some organisms may temporarily survive harsh conditions by forming resistant stages (spores) or through specific mechanisms (heavy metal resistance), true extremophiles require these conditions.” (Prieur, 2015).

Habitable zone: “The circumstellar habitable zone (HZ) is defined as the annulus around a main sequence star where a rocky planet similar to Earth in composition and mass with an atmosphere can support liquid water on its surface. […] The width and distance of this annulus, using this definition, depend mainly on the stellar luminosity and spectral energy distribution. A planet in the habitable zone is not necessarily habitable.” (Kaltenegger & Segura, 2015).

Technosignatures: “… potentially detectable signatures and signals of the presence of distant advanced civilizations.” (Kaufman, 2018).

According to the *Astrobiology Primer 2.0* (Domagal-Goldman & Wright et al, 2016 - adapted for concision and clarity), currently known conditions and ingredients for life are:

- Energy: A source of energy is necessary for metabolism and to maintain the functions carried out by a cell. (...) Life on Earth uses either solar energy or chemical energy sources that include both organic (carbon) compounds and inorganic elements and molecules.
- Common essential elements: All known Earth life uses the same main set of elements to form biomolecules (Carbon, Hydrogen, Nitrogen, Oxygen, Phosphorus and Sulfur) and shares a common carbon-based biochemistry. Carbon is an excellent element from which to build life, because carbon forms macromolecules with a variety of other elements and itself. (...)
- Solvent: A solvent to host chemical interactions is considered a universal requirement for all life. (...) Many astrobiologists consider water to be the most probable solvent for life on any planet.

1. **Identification**
2. Coders:

1 = Coder A; 2 = Coder B; 3 = Coder C

1. Set number (combination of paper, press release and news article identification):
2. Type of document:

1 = Research paper; 2 = Press release; 3 = News article

1. If 3, code the newspaper in which the story was published:

1 = The New York Times; 2 = The Guardian; 3 = USA Today (pilot study); 4 = The Independent (pilot study); 5 = Estadão; 6 = Folha de S. Paulo; 7 = Público; 8 = El País

1. Date of publication (day/month/year):
2. Author(s) or news agency (e.g. Reuters, Associated Press, Agence France-Presse) or the name of the research institution (0 if none):
3. Title/headline:
4. Length of the article (word count):

**II. Research characterization**

Here, the goal is to obtain data that may or may not indicate which of these broad sub-areas are more prone to misrepresentations, speculations and promises.

1. Astrobiology-related broad subarea (if more than one, code the most prominent):

1 = Solar System planetary science (Solar System planets and moons, asteroids, comets).

2 = Extrasolar astronomy (exoplanets, stellar-planetary formation, protoplanetary disks, interstellar medium).

3 = Origins of life and alternative biochemistries (abiogenesis, early Earth biosphere, extremophiles, non-water solvents, non-carbon-based life).

4 = SETI (Search for Extraterrestrial Intelligence, technosignatures).

5 = Other(s): ______________________________

**III. Speculations about the SLE and its research results**

According to the Cambridge Dictionary, speculation is “the activity of guessing possible answers to a question without having enough information to be certain”. The goals are: 1. to detect speculations about the search for life elsewhere and the research results related to the SLE; and 2. to capture any significant variations between the paper, the press release and the news article regarding these speculations.

We will try to capture 5 (five) kinds of **speculations** related to the SLE:

**A. speculations about the *outcomes* of the SLE (speculations about *the prospects of life detection* or speculations about *answering the question of whether there is life elsewhere*).**

Examples of **A**: “New study finds the chances of uncovering life on Mars are better than previously expected”; “Nasa could find alien planets that host life after new oxygen breakthrough”.

**B. speculations about the *significance of a particular research result* for the SLE (speculations about the result being a step toward the detection of life elsewhere, or speculations about the result being a step forward to understand the conditions and ingredients for life elsewhere, or speculations about the result being a step forward to understand the prevalence of life in the universe).**

Examples of **B**: “The new molecular system will allow scientists who are looking for life elsewhere in the universe to re-think what they are actually looking for, researchers said.”; “Now scientists hope that the new technique could allow them to spot the same signal coming from other planets, and in so doing perhaps spot planets that might be home to alien life.”

**C. speculations about the *evidential status of a research result* (speculations about the likelihood that a particular research result constitutes *evidence* of the existence of life beyond Earth).**

Example of **C**: **“**Nasa's James Webb Space Telescope may have discovered tentative evidence of a sign of life on a faraway planet.”

**D. speculations about the *existence of life beyond Earth*.**

Examples of **D**: “As for Venus, there’s always the possibility that any life in the planets’ clouds – if it exists – could be totally unlike anything on Earth and adapted to the hothouse planet’s extremely hot and harsh conditions, according to scientists.”; “Life on Mars? Researchers find signs of rivers on Mars, an indicator of potential past life”.

**E. speculations about *conditions and/or ingredients for the existence/emergence of life beyond Earth.***

Examples of **E**: “On Earth, river corridors are so important for life, chemical cycles, nutrient cycles and sediment cycles. Everything is pointing to these rivers behaving similarly on Mars. It offers a vision of Mars where most of the planet once had the right conditions for life.”; “NASA says its planet-hunting telescope has found 10 new planets outside our solar system that are likely the right size and temperature to potentially have life on them.”

1. **(A)** Are there **speculations** about the ***outcomes* of the SLE**?

0 = no; 1 = yes

**Instruction to coders:** Here we are looking for the presence of speculations about *the prospects of life detection* or speculations about *the prospects of answering the question of whether there is life elsewhere*. A speculation of this kind may be construed as a conjecture on whether life beyond Earth is (or is not) likely to be found, or as a conjecture about the likelihood that scientific research will find answers for the question of whether there is life elsewhere.

If 1, code questions 11 and 12. If 0, go to question 13:

1. **(A)** Regarding the ***outcomes* of the SLE** (speculations about *the prospects of life detection* or speculations about *the prospects of answering the question of whether there is life elsewhere*), what is the approximate level of the speculation? (In case different levels of speculation of this kind appear, code the highest level you find.)

0 = it’s impossible (to detect life elsewhere or to answer the question)

1 = it’s unlikely (to detect life elsewhere or to answer the question)

2 = it’s possible/plausible (to detect life elsewhere or to answer the question)

3 = it’s likely (to detect life elsewhere or to answer the question)

4 = it’s inevitable (to detect life elsewhere or to answer the question)

5 = it has probably been found already/it has probably been answered

Fictitious examples:

0: “We will never find life beyond Earth”, stated Dwight Schrute, assistant professor at the University of New Atlantis.

1: “Life beyond Earth may exist, but it is like searching for a needle in the haystack and the search for it is unlikely to ever yield any positive results”, said Pamela Beasley, astrophysicist at the New Atlantis Observatory.

2: Angela Martin, a researcher at the New Atlantis University, says the detection of life beyond Earth is a plausible outcome of new space missions focused on Venus.

3: Darryl Philbin, the leading author of the paper, is confident the search for biosignatures in exoplanets' atmospheres is likely to produce robust evidence of life beyond Earth in the next decade.

4: “We are bound to find life beyond Earth, it’s just a matter of time”, told us the researcher Creed Bratton, from the New Atlantis Institute of Astrobiology.

5: Kelly Kapoor, astrobiologist at the University of New Atlantis, claimed that we already have proof of the existence of life beyond Earth, we just have to interpret it in the right way.

1. **(A) (Press releases and news articles)** Where are the speculations?

12.1 = in the title/subtitle: 0 = no, 1 = yes

12.2 = in the body of the text, it is not in quotes and it is not attributed to an expert: 0 = no, 1 = yes

12.3 = in quotes of the author(s) of the paper, or it is attributed to the author(s): 0 = no, 1 = yes

12.4 = in quotes of an outside expert (a scientist who did not participate in the research), or it is attributed to an outside expert: 0 = no, 1 = yes

12.5 = other: 0 = no, 1 = yes (annotate on the cell of the spreadsheet)

**Instructions to coders:** in 12.5 (**and every other XX.5**) you should code as **1** (and annotate on the cell of the spreadsheet) every time that the speculation or promise is attributed to an institution (e.g. NASA, universities, etc.) or to “scientists” in a general, unspecific way, or even to “the scientific community”. Be careful: sometimes the words “scientists” or “experts” are used in a general, unspecified way but context shows that they reference the authors of the study or specific outside experts; in this case, the XX.5 question should be answered with a 0 if there is no general, unspecified attribution of information to “scientists” or “experts”.

1. **(B)** Are there speculations about **the significance of a particular research result for the SLE**?

0 = no; 1 = yes

**Instructions to coders:** Here we are looking for **speculations** about the **importance and/or meaning of a research result** **for the SLE**. It can assume the form of a reflection on what the research being presented might entail for the SLE. The result can be construed as a refinement of methods and approaches for the detection of life elsewhere, or as a way of narrowing down the scope of the search for the conditions and ingredients for life elsewhere, or as an improvement of estimates of the prevalence of life in the universe. This is **not** designed to capture when a particular research result is (or is not) significant for the SLE but **when speculations are made** **about the meaning and the importance of the result for the SLE**. Of course speculations about the significance and the significance itself may overlap but we are **looking for the speculations here**.

If 1, code questions 14 and 15. If 0, go to question 16:

1. **(B)** Regarding the **speculations about the significance of a particular research result for the SLE**, what is the approximate nature of the speculations?

0 = setback – the results are a setback to the goal of detecting life elsewhere, or to have a better understanding of life’s prevalence in the universe and/or the conditions and ingredients for the existence of life beyond Earth

1 = neutral – the results are neither a setback nor an advancement to the goal of detecting life elsewhere, or to have a better understanding of life’s prevalence in the universe and/or the conditions and ingredients for the existence of life beyond Earth

2 = advance – the results are a step forward in the goal of detecting life elsewhere, or to have a better understanding of life’s prevalence in the universe and/or the conditions and ingredients for the existence of life beyond Earth

3 = game changing and/or revolutionary – the results are potentially revolutionary, changing our understanding of life’s prevalence in the universe or of the conditions for the existence of life beyond Earth

4 = other/unsure (quote speculation in note on the cell of the spreadsheet)

Fictitious examples:

0: “This result is puzzling and nobody knows if we might be searching in the wrong places for life beyond Earth”, said Toby Flenderson, astrobiologist at the University of New Atlantis.

1: “Our study shows that we still have not advanced our understanding of life’s prevalence in the universe”, pondered Jim Halpert, professor at the University of New Atlantis.

2: “This result does not mean that life exists there, but it makes life more plausible and that will inform future searches in similar planets” said Philys Vance, lead author of the paper.

3: Michael Scott, the director of the New Atlantis Astrobiology Institute, said the result has the potential to completely change our definitions of life and how we look for it beyond Earth. “What we’re doing is revolutionary”, he claims.

1. **(B) (Press releases and news articles)** Where are the speculations?

15.1 = in the title/subtitle: 0 = no, 1 = yes

15.2 = in the body of the text, it is not in quotes and it is not attributed to an expert: 0 = no, 1 = yes

15.3 = in quotes of the author(s) of the paper, or it is attributed to the author(s): 0 = no, 1 = yes

15.4 = in quotes of an outside expert (a scientist who did not participate in the research), or it is attributed to an outside expert: 0 = no, 1 = yes

15.5 = other: 0 = no, 1 = yes (annotate on the cell of the spreadsheet)

1. **(C)** Are there **speculations** about the **evidential status of a research result**?

0 = no; 1 = yes

**Instruction to coders:** Here we are looking for speculations about whether a particular research result may **constitute *evidence* of life beyond Earth**. We are **not** judging the evidential status of a research result but looking for **speculations about its evidential status**. Is it being construed as a potential proof of life elsewhere? Is it being portrayed as evidence that points to the existence of life beyond Earth? If the answer for questions like these is yes, then code 1.

If 1, code questions 17 and 18. If 0, go to question 19:

1. **(C)** Regarding the **evidential status of a research result**, what is the approximate character of the speculations? (In case different levels of speculation of this kind appear, code the highest level you find.)

1 = the result may be evidence of life beyond Earth, but there are or there might be **other non-biological** explanations for it

2 = the result may be evidence of life beyond Earth, and life is the **best** available explanation for it

3 = the result may be evidence of life beyond Earth, and life is the **only** available explanation for it

4 = the result **is** evidence of life beyond Earth

Fictitious examples:

1: “We’ve found signs that could indicate biological activity in this atmosphere, but abiogenic processes could have caused it”, said Stanley Hudson, professor at the University of New Atlantis.

2: “We have competing explanations for the observed phenomena, but in my view, biological activity is the best available hypothesis”, said Andy Bernard, from the Cornell Astrobiology Institute.

3: Ryan Howard, professor of astrobiology at the University of New Atlantis, said the result can only be explained by biological processes, but more funding is needed to test the hypothesis.

4: “The result is robust evidence of the existence of life beyond Earth”, said Kevin Malone, astrobiologist at the New Atlantis Astrobiology Institute.

1. **(C) (Press releases and news articles)** Where are the speculations?

18.1 = in the title/subtitle: 0 = no, 1 = yes

18.2 = in the body of the text, it is not in quotes and it is not attributed to an expert: 0 = no, 1 = yes

18.3 = in quotes of the author(s) of the paper, or it is attributed to the author(s): 0 = no, 1 = yes

18.4 = in quotes of an outside expert (a scientist who did not participate in the research), or it is attributed to an outside expert: 0 = no, 1 = yes

18.5 = other: 0 = no, 1 = yes (annotate on the cell of the spreadsheet)

1. **(D)** Are there **speculations** about the **existence of life beyond Earth**?

0 = no; 1 = yes

**Instruction to coders:** Here we are looking for speculations about the existence of life beyond Earth. It encompasses present, past or future existence of extraterrestrial life - in general or in a particular environment. It also encompasses speculations about alternative life forms beyond Earth and the possible diversity of life in the universe. Speculations about the existence of life and the existence of conditions and/or ingredients for life may sometimes overlap in the same sentence. References for the possible existence of life elsewhere that doesn’t mention the word life include mentions of living beings, biospheres, biological activity, biogenicity of a signal, and similar/derived terms and expressions.

If 1, code questions 20 and 21. If 0, go to question 22.

1. **(D)** Regarding the **existence of life beyond Earth**, what is the approximate nature of the speculation? (In case different levels of speculation of this kind appear, code the highest level you find.)

0 = it’s impossible (“there is no life beyond Earth”)

1 = it’s unlikely (“life is unlikely to exist beyond Earth”)

2 = it’s possible/plausible (“there could be life beyond Earth in general or in a particular environment elsewhere”)

3 = it’s likely (“life probably exists beyond Earth in general or in a particular environment elsewhere”)

4 = it’s a certainty (“there is life beyond Earth in general or in a particular environment elsewhere”)

1. **(D) (Press releases and news articles)** Where are the speculations?

21.1 = in the title/subtitle: 0 = no, 1 = yes

21.2 = in the body of the text, it is not in quotes and it is not attributed to an expert: 0 = no, 1 = yes

21.3 = in quotes of the author(s) of the paper, or it is attributed to the author(s): 0 = no, 1 = yes

21.4 = in quotes of an outside expert (a scientist who did not participate in the research), or it is attributed to an outside expert: 0 = no, 1 = yes

21.5 = other: 0 = no, 1 = yes (annotate on the cell of the spreadsheet)

1. **(E)** Are there **speculations** about **conditions and/or ingredients for the existence/emergence of life beyond Earth?**

0 = no; 1 = yes

**Instructions to coders:** Here we are looking for speculations about the existence of conditions and/or ingredients for life beyond Earth. It encompasses speculations about the past, present or future existence of conditions and/or ingredients for life beyond Earth - in general or in a particular environment. It encompasses speculations about the existence of necessary conditions and/or sufficient conditions for life beyond Earth (the distinction between sufficient and necessary conditions for life beyond Earth is frequently blurred or absent in public discourse). Speculations about the existence of life and the existence of conditions and/or ingredients for life may sometimes overlap in the same sentence. Speculations for the conditions and/or ingredients for life don't have to include all the sufficient conditions for life to count as a positive answer, **but the mere mention for the existence of a necessary condition (e.g. liquid water, organic molecules) does not constitute a speculation in itself** (although it is sometimes associated to a speculation of this kind). About the circumstellar habitable zone: speculations on the habitability of other (exo)planets and (exo)moons should link the habitable zone to the prospects of habitability and the existence of conditions for life. The mere mention of the habitable zone by itself does not constitute a speculation. Also, we know that some necessary ingredients for life exist beyond Earth (e.g. complex organic molecules, liquid water) and merely stating that doesn’t count as a speculation, but it is a speculation if the statement tries to fill the gap in knowledge about the existence of necessary ingredients and sufficient life supporting conditions.

If 1, code questions 23 and 24. If 0, go to question 25.

1. **(E)** Regarding the **conditions and/or ingredients for life beyond Earth**, what is the approximate nature of the speculation? (In case different levels of speculation of this kind appear, code the highest level you find.)

0 = it’s impossible (“there are no conditions and/or ingredients for life beyond Earth”)

1 = it’s unlikely (“conditions and/or ingredients for life beyond Earth are unlikely to exist”)

2 = it’s possible/plausible (“there could be conditions and/or ingredients for life beyond Earth or in a particular environment elsewhere”)

3 = it’s likely (“conditions and/or ingredients for life beyond Earth in general or in a particular environment elsewhere are likely to exist”)

4 = it’s a certainty (“conditions and/or ingredients for life beyond Earth do exist in general or in a particular environment elsewhere”)

1. **(E) (Press releases and news articles)** Where are the speculations?

24.1 = in the title/subtitle: 0 = no, 1 = yes

24.2 = in the body of the text, it is not in quotes and it is not attributed to an expert: 0 = no, 1 = yes

24.3 = in quotes of the author(s) of the paper, or it is attributed to the author(s): 0 = no, 1 = yes

24.4 = in quotes of an outside expert (a scientist who did not participate in the research), or it is attributed to an outside expert: 0 = no, 1 = yes

24.5 = other: 0 = no, 1 = yes (annotate on the cell of the spreadsheet)

**IV. Promises and expectations**

The meaning of promise used in this section is related to expectation and optimism. From the Cambridge Dictionary: “the idea that someone or something is likely to develop successfully and that people expect this to happen”.

In this section, the goal is to understand: 1. **how often** the science communication ecosystem makes or circulates promises and expectations of the SLE, and 2. the **nature** of promises and expectations of the SLE that most frequently circulate in the public discourse. Also, we should detect any significant variations between the original paper, the press release and the news article on that. That could yield data for further in depth analysis about the role of scientists, institutions, journalists and media outlets in hyping up (fostering high and positive expectations about) the SLE in the public discourse.

Expectations about the SLE seemed to shift from “if there is life beyond Earth, how can we discover it?” to “when we detect clues that could be interpreted as evidence of life elsewhere, we have to make sure we have a robust procedure of interpretation and assessment so that we adequately communicate these results to the scientific community and the public”. An analysis on the recently proposed CoLD Scale (Green et al, 2021) for communication of life detection results criticized the initiative precisely on that, arguing that “the goal has shifted from exploration, where we accept what we do or do not find, to finding life” (Lenardic et al, 2023).

Circulating in the public discourse about astrobiology there are at least 3 (three) distinguishable kinds of promises and expectations related to the SLE for the foreseeable future:

1. **The SLE is expected to detect life beyond Earth or produce evidence pointing to its existence.**

Example of **A**: “There will be "strong indications" of alien life within a decade and "definite evidence" of it within 20 to 30 years, NASA's chief scientist has said.”

1. **The SLE is making or will make progress. (This includes the search for life itself and the search for conditions/ingredients for life.)**

Examples of **B**: “Scientists have developed a new method for detecting oxygen in exoplanet atmospheres that may accelerate the search for life.”; “This opens up exciting new avenues for future exploration, potentially leading us closer to answering the age-old question: are we alone in the universe?”; "’This intriguing, distant world gives us even greater hope that a second Earth lies among the stars, waiting to be found,’ said Thomas Zurbuchen, associate administrator of NASA's Science Mission Directorate in Washington. ‘The data gathered by missions like Kepler and our Transiting Exoplanet Survey Satellite [TESS] will continue to yield amazing discoveries as the science community refines its abilities to look for promising planets year after year.’”

1. **Technological development will/may provide clues, evidence or answers for the SLE (this includes the search for life itself and the search for conditions/ingredients for life).**

Examples of **C**: “The newest spaceborne instruments, including those onboard NASA’s James Webb Space Telescope, are designed not just to detect these distant worlds, but to reveal some of their characteristics. That includes the composition of their atmospheres, which could offer clues to the possible presence of life.”; “‘That strengthens the probability that, if life ever evolved on Mars, this will be revealed in future missions.’ Missions such as Nasa Mars sample return, which will bring samples of Martian soil taken by the space agency’s Perseverance rover back to Earth in the early 2030s.”

1. Is a promise/expectation of the kind **A** **(the SLE is expected to detect life beyond Earth or produce evidence pointing to its existence)** present?

0 = no; 1 = yes

**Instruction to coders:** Here we are looking for expressions of expectations that scientific research will detect life beyond Earth or produce evidence pointing to its existence some time in the future.

If 1, code question 26. If 0, go to question 27.

1. **(Press releases and news articles)** Where is the promise/expectation **A** being made?

26.1. in the title/subtitle: 0 = no, 1 = yes

26.2. in the body of the text, it is not in quotes and it is not attributed to an expert: 0 = no, 1 = yes

26.3. in quotes of the author(s) of the paper, or it is attributed to the author(s): 0 = no, 1 = yes

26.4. in quotes of an outside expert (a scientist that did not participate in the research), or it is attributed to an outside expert: 0 = no, 1 = yes

26.5. other: 0 = no, 1 = yes (annotate on the cell of the spreadsheet)

1. Is a promise/expectation of the kind **B (the SLE is making incremental progress or will make progress)** present?

0 = no; 1 = yes

**Instruction to coders:** Here our goal is to detect the promise of progress of the SLE, which is constituted by the expectation that the SLE is getting nearer to its goals of finding life elsewhere and/or understanding the prevalence of life in the universe. **We are not looking into the result itself, trying to distinguish which result constitutes progress and which one does not.** It is not the job of the coder to interpret if the result can be considered a progress or to interpret if the SLE is making progress – either in a general way or because of a particular research result. The job of the coder is to find if there is a promise or an expectation being formulated about the development of the SLE. **It is usually expressed in a way that conveys a picture of progress – it implies that the SLE is getting nearer to its goals, or it’s going in the right direction.** It can also be construed as acceleration of the development of the SLE.

If 1, code question 28. If 0, go to question 29.

1. **(Press releases and news articles)** Where is the promise/expectation **B** being made?

28.1. in the title/subtitle: 0 = no, 1 = yes

28.2. in the body of the text, it is not in quotes and it is not attributed to an expert: 0 = no, 1 = yes

28.3. in quotes of the author(s) of the paper, or it is attributed to the author(s): 0 = no, 1 = yes

28.4. in quotes of an outside expert (a scientist that did not participate in the research), or it is attributed to an outside expert: 0 = no, 1 = yes

28.5. other: 0 = no, 1 = yes (annotate on the cell of the spreadsheet)

1. Is a promise/expectation of the kind **C** **(technological development will/may provide clues, evidence or answers for the SLE)** present?

0 = no; 1 = yes

**Instruction to coders:** Here we are looking for the expectation that progress in technologies related to scientific research will or may provide clues, evidence or answers for the SLE. This includes expectations about still inexistent and unforeseeable technologies. **It is also not limited to future technologies or future technological development**: it may refer to present day technology, as well as what was, at some point in time, a future technology but it’s now current technology (e.g. James Webb Space Telescope). It may also refer to what was already present day technology at some point in time (e.g. ALMA in 2018, Hubble in 1998). It could also be construed as a situation in which hypothetical future space exploration missions would be necessary to settle questions about the existence of life elsewhere or the conditions for its existence. The focus here is the optimism about using future or present day technology to obtain answers for the SLE.

If 1, code question 30.

1. **(Press releases and news articles)** Where is the promise/expectation **C** being made?

30.1. in the title/subtitle: 0 = no, 1 = yes

30.2. in the body of the text, it is not in quotes and it is not attributed to an expert: 0 = no, 1 = yes

30.3. in quotes of the author(s) of the paper, or it is attributed to the author(s): 0 = no, 1 = yes

30.4. in quotes of an outside expert (a scientist that did not participate in the research), or it is attributed to an outside expert: 0 = no, 1 = yes

30.5. other: 0 = no, 1 = yes (annotate on the cell of the spreadsheet)

**Table: the codebook in a nutshell.**

| **Speculations and promises/expectations** | **Category** | **Example from outside the corpus** |
| --- | --- | --- |
| **Speculations** | ***Outcomes***  Speculations about the *outcomes* of the SLE | “New study finds the chances of uncovering life on Mars are better than previously expected” |
|  | ***Significance***  Speculations about the *significance* of a particular research result for the SLE | “Now scientists hope that the new technique could allow them to spot the same signal coming from other planets, and in so doing perhaps spot planets that might be home to alien life.” |
|  | ***Evidence***  Speculations about the *evidential status* of a research result | **“**Nasa's James Webb Space Telescope may have discovered tentative evidence of a sign of life on a faraway planet.” |
|  | ***Existence***  Speculations about the *existence of life* beyond Earth | “Life on Mars? Researchers find signs of rivers on Mars, an indicator of potential past life” |
|  | ***Conditions***  Speculations about *conditions and/or ingredients for the existence/emergence of life* beyond Earth | “On Earth, river corridors are so important for life, chemical cycles, nutrient cycles and sediment cycles. Everything is pointing to these rivers behaving similarly on Mars. It offers a vision of Mars where most of the planet once had the right conditions for life.” |
| **Promises/Expectations** | ***Detection***  The SLE is *expected to detect life* beyond Earth or *produce evidence* pointing to its existence | “There will be "strong indications" of alien life within a decade and "definite evidence" of it within 20 to 30 years, NASA's chief scientist has said.” |
|  | ***Progress***  The SLE is making or will make *progress* | “This opens up exciting new avenues for future exploration, potentially leading us closer to answering the age-old question: are we alone in the universe?” |
|  | ***Technology***  *Technological development* will/may provide clues, evidence or answers for the SLE | “‘That strengthens the probability that, if life ever evolved on Mars, this will be revealed in future missions.’ Missions such as Nasa Mars sample return, which will bring samples of Martian soil taken by the space agency’s Perseverance rover back to Earth in the early 2030s.” |

**References:**

Domagal-Goldman & Wright, et al. (2016). The Astrobiology Primer v2.0. Astrobiology. Aug;16(8):561-653. doi: 10.1089/ast.2015.1460.

Gargaud, M., et al. (2020). Encyclopedia of Astrobiology. Springer, Berlin, Heidelberg. <https://doi.org/10.1007/978-3-662-44185-5_100118>

Green, J., Hoehler, T., Neveu, M. et al. Call for a framework for reporting evidence for life beyond Earth. Nature 598, 575–579 (2021). <https://doi.org/10.1038/s41586-021-03804-9>

Javaux, E.J. (2015). Biomarkers. In: Gargaud, M., et al. Encyclopedia of Astrobiology. Springer, Berlin, Heidelberg. <https://doi.org/10.1007/978-3-662-44185-5_180>

Kaltenegger, L., Segura, A. (2015). Habitable Zone. In: Gargaud, M., et al. Encyclopedia of Astrobiology. Springer, Berlin, Heidelberg. <https://doi.org/10.1007/978-3-662-44185-5_685>

Kaufman, M. (2018). Technosignatures and the Search for Extraterrestrial Intelligence. [NASA/Astrobiology website](https://astrobiology.nasa.gov/news/technosignatures-and-the-search-for-extraterrestrial-intelligence/).

Latham, D.W., Haghighipour, N. (2015). Exoplanets, Discovery. In: Gargaud, M., et al. Encyclopedia of Astrobiology. Springer, Berlin, Heidelberg. <https://doi.org/10.1007/978-3-662-44185-5_552>

Lenardic, A., Seales, J., Moore, W.B. et al. Communicating astrobiology in words not numbers and with facts not fiction. Nat Astron 7, 1009 (2023). <https://doi.org/10.1038/s41550-023-02031-8>

McLoughlin, N. (2015). Biogenicity. In: Gargaud, M., et al. Encyclopedia of Astrobiology. Springer, Berlin, Heidelberg. <https://doi.org/10.1007/978-3-662-44185-5_171>

Millar, N. et al. (2022). Trends in the Use of Promotional Language (Hype) in Abstracts of Successful National Institutes of Health Grant Applications, 1985-2020. JAMA Netw Open. 2022;5(8):e2228676. doi:10.1001/jamanetworkopen.2022.28676

Prieur, D. (2015). Extremophiles. In: Gargaud, M., et al. Encyclopedia of Astrobiology. Springer, Berlin, Heidelberg. <https://doi.org/10.1007/978-3-662-44185-5_568>

Ross PT, Bibler Zaidi NL. Limited by our limitations. Perspect Med Educ. 2019 Aug;8(4):261-264. doi: 10.1007/s40037-019-00530-x. PMID: 31347033; PMCID: PMC6684501.

Tirard, S. (2015). Abiogenesis. In: Gargaud, M., et al. Encyclopedia of Astrobiology. Springer, Berlin, Heidelberg. <https://doi.org/10.1007/978-3-662-44185-5_2>

W.E. Walker, P. et al. (2003) Defining Uncertainty: A Conceptual Basis for Uncertainty Management in Model-Based Decision Support, Integrated Assessment, 4:1, 5-17, DOI: 10.1076/iaij.4.1.5.16466
