# Supplementary material for: Communicating astrobiology and the search for life elsewhere: Speculations and promises of a developing scientific field in newspapers, press releases and papers
Source: PLoS One. 2025 Jul 29;20(7):e0328766. doi: 10.1371/journal.pone.0328766 (PMC12306777; doi:10.1371/journal.pone.0328766)
Supplement: S3 Appendix — (DOCX) [file pone.0328766.s003.docx]

**Appendix III – Full intercoder reliability results**

**Pilot studies**

Two pilot studies were conducted to develop the categories, the questions and instructions of the codebook, to ensure its objectivity and applicability by different coders. The samples coded in each of the pilot studies were selected and searched for with the same criteria used for the actual analyzed corpus. The first pilot study examined 11 news articles from the American newspaper USA Today and their respective 11 papers and 12 press releases (one of the sets had two different press releases). The second pilot study examined 11 news articles from the British newspaper The Independent, along with 11 press releases and 11 papers. To build both pilot studies corpora we used the same procedure and keywords used for the actual corpus. The number of articles selected for both pilot studies corpora was to get safely 10% of what would be the final number of sets in the English corpus (at the time of the pilot study, 70 sets were completed. The final corpus had 80 sets with English news stories).

The first pilot study showed the need for improvement of instructions for coders, specifically in speculations about the significance of research results and promises/expectations about progress in the SLE. Because of an initial lack of clarity in the description of these categories, there were substantial disagreements between coders. Two categories, the evidence speculation and the detection promise, did not occur. Other categories, like the existence speculation and the technology promise presented acceptable/substantial intercoder reliability. In all of the categories, clearer and explicit instructions for coders were added to the codebook after discussion between coders. After the addition of these instructions and additional discussions, results from the second pilot study showed a substantial improvement in the intercoder reliability for the problematic categories. During this phase, both coders examined the disagreements that were identified as coding error or clear misinterpretation. These disagreements were solved through discussions and in-depth analysis by both coders. After these adjustments, all of the main categories had ICR results above sufficient, although the coded levels of speculative content were still unreliable.

**Intercoder reliability results**

To check the intercoder reliability of the coding scheme applied to the actual corpus in English, we randomly selected a sample of 14 news articles (11.8% of the total), 21 press releases (12.3%) and 21 papers (11.1%). After the sample was coded, coders discussed the disagreements, coding errors and clear misinterpretations. The review process and analysis of problematic cases of disagreements in the coding of the sample, with subsequent adjustments agreed upon by both coders, resulted in substantial agreement between coders, with an average of Krippendorff’s alpha 0.819 for main categories and 0.825 for internal questions about the attribution of the speculation and promises/expectations. Intercoder reliability was also checked for Portuguese and Spanish language corpora, but with a different second coder. A randomly selected sample of 12 news stories in Portuguese (10.1% of the total) and 5 news stories in Spanish (13.8%) was coded. After the same process of review applied to the English corpus, the average Krippendorff’s alpha in the Portuguese/Spanish corpus for the main categories was 0.811 and 0.856 for the internal questions. The coded levels of speculative content were not sufficiently reliable to be included in the results.

**ICR results - English corpus sample**

Average Krippendorff's alpha for the 5 main questions in which it was possible to have a measurement of agreement (2 categories were non occurring): **0.819**

Average Krippendorff's alpha for the internal questions (“where”) in which it was possible to have a measurement of agreement: **0.825**

**10: Krippendorff's alpha: 0.661**

11: No variation, therefore no reliability coefficients could be calculated.

12.1: No variation.

12.2: No variation.

12.3: No variation.

12.4: No variation

12.5: No variation.

**13: Krippendorff's alpha: 0.855**

14: No variation.

15.1: No variation.

15.2: Krippendorff's alpha: 1

15.3: Krippendorff's alpha: 0.593 (only one disagreement)

15.4: Krippendorff's alpha: 0.686 (only one disagreement)

15.5: No variation.

**16: Only one occurrence. No variation.**

17: Only one occurrence. No variation.

18.1: No occurrence in the sample.

18.2: No occurrence in the sample.

18.3: No occurrence in the sample.

18.4: No occurrence in the sample.

18.5: No occurrence in the sample.

**19: Krippendorff's alpha: 0.827**

20: Krippendorff's alpha (ordinal): 1

21.1: Krippendorff's alpha: 0.837

21.2: Krippendorff's alpha: 0.702

21.3: Krippendorff's alpha: 1

21.4: Krippendorff's alpha: 0.812

21.5: Krippendorff's alpha: 1

**22: Krippendorff's alpha: 0.823**

23: Krippendorff's alpha (ordinal): 0.784

24.1: Krippendorff's alpha: 0.605

24.2: Krippendorff's alpha: 0.574

24.3: Krippendorff's alpha: 0.826

24.4: Krippendorff's alpha: 0.866

24.5: Krippendorff's alpha: 1

**25: No occurrence in the sample.**

26.1: No occurrence in the sample.

26.2: No occurrence in the sample.

26.3: No occurrence in the sample.

26.4: No occurrence in the sample.

26.5: No occurrence in the sample.

**27: Krippendorff's alpha: 0.888**

28.1: No variation.

28.2: Krippendorff's alpha: 1

28.3: Krippendorff's alpha: 1

28.4: Krippendorff's alpha: 1

28.5: No variation.

**29: Krippendorff's alpha: 0.862**

30.1: No variation.

30.2: Krippendorff's alpha: 0.808

30.3: Krippendorff's alpha: 0.791

30.4: Krippendorff's alpha: 1

30.5: No variation.

**ICR results - Portuguese/Spanish corpus sample**

Average Krippendorff's alpha for main questions: **0.811** (question 25 with 100% did not enter calculation of average).

Internal questions average Krippendorff's alpha: **0.856** (those that exhibited no variation/measure of agreement were not part of the average).

**10: Krippendorff's alpha: 0.645**

11: Krippendorff's alpha (ordinal): 0

12.1: No variation, therefore no reliability coefficients could be calculated.

12.2: No variation.

12.3: No variation.

12.4: No variation.

12.5: No variation.

**13: Krippendorff's alpha: 0.87**

14: Krippendorff's alpha (ordinal): 0.083

15.1: Krippendorff's alpha: 1

15.2: Krippendorff's alpha: 0

15.3: Krippendorff's alpha: 1

15.4: Krippendorff's alpha: 1

15.5: No variation.

**16: Krippendorff's alpha: 0.645**

17: Krippendorff's alpha (ordinal): 0

18.1: No variation.

18.2: No variation.

18.3: No variation.

18.4: No variation.

18.5: No variation.

**19: Krippendorff's alpha: 0.879**

20: Krippendorff's alpha (ordinal): 0

21.1: Krippendorff's alpha: 1

21.2: No variation.

21.3: Krippendorff's alpha: 0.64

21.4: No variation.

21.5: No variation.

**22: Krippendorff's alpha: 0.884**

23: Krippendorff's alpha (ordinal): 1

24.1: Krippendorff's alpha: 0.779

24.2: Krippendorff's alpha: 1

24.3: Krippendorff's alpha: 1

24.4: Krippendorff's alpha: 1

24.5: No variation.

**25: 100% (17/0)**

26.1: No variation.

26.2: No variation.

26.3: No variation.

26.4: No variation.

26.5: No variation.

**27: Krippendorff's alpha: 0.879**

28.1: No variation.

28.2: Krippendorff's alpha: 0.686

28.3: Krippendorff's alpha: 0.593

28.4: Krippendorff's alpha: 1

28.5: No variation.

**29: Krippendorff's alpha: 0.879**

30.1: No variation.

30.2: Krippendorff's alpha: 1

30.3: Krippendorff's alpha: 1

30.4: Krippendorff's alpha: 1

30.5: No variation.
