# Supplementary figures and images for: Communicating astrobiology and the search for life elsewhere: Speculations and promises of a developing scientific field in newspapers, press releases and papers
Source: PLoS One. 2025 Jul 29;20(7):e0328766. doi: 10.1371/journal.pone.0328766 (PMC12306777; doi:10.1371/journal.pone.0328766)

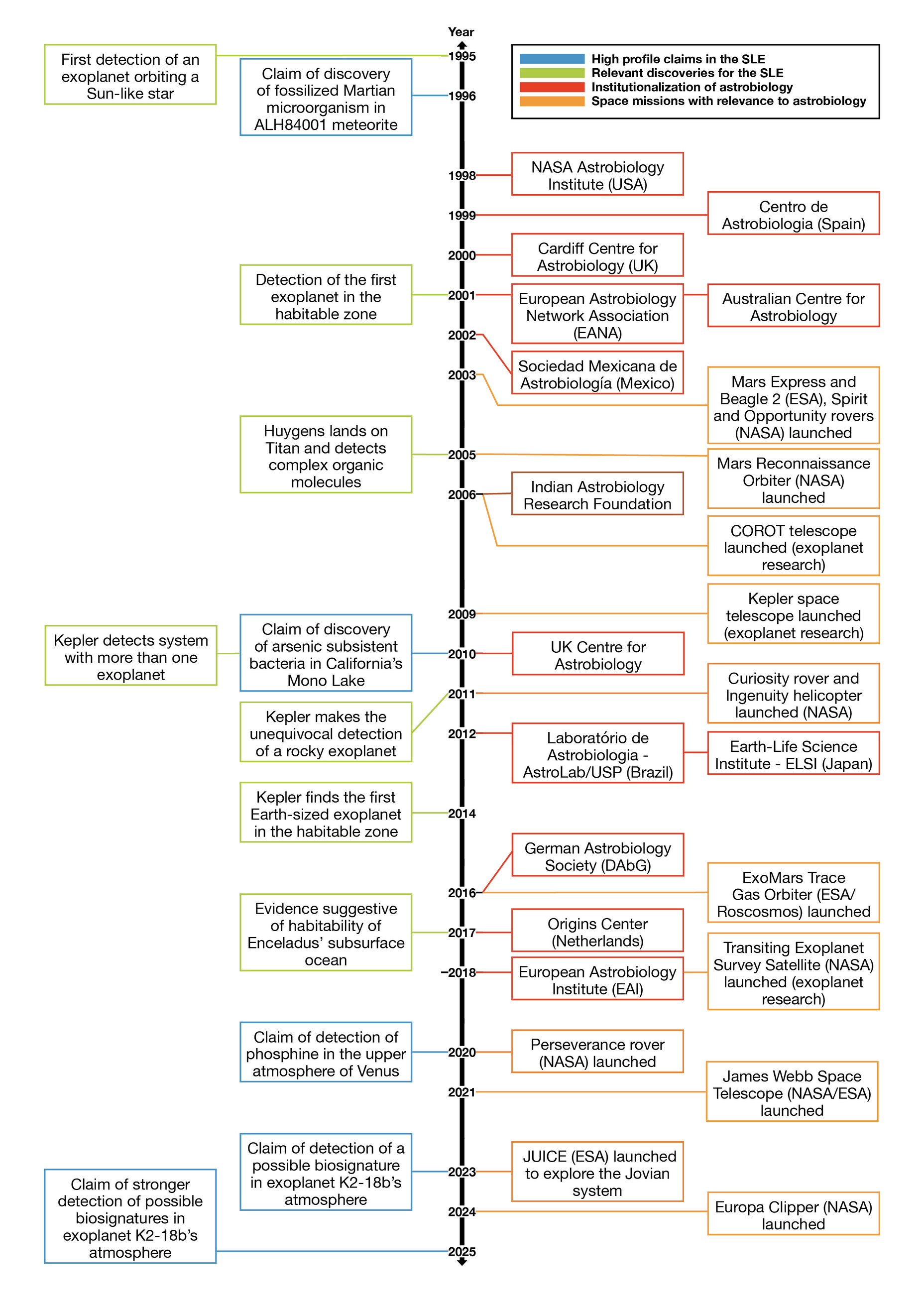

Supplement: S5 Fig — (TIF) [file pone.0328766.s005.tif]
